# Molecular Evidence of Adenosine Deaminase Linking Adenosine A_2A_ Receptor and CD26 Proteins

**DOI:** 10.3389/fphar.2018.00106

**Published:** 2018-02-15

**Authors:** Estefanía Moreno, Júlia Canet, Eduard Gracia, Carme Lluís, Josefa Mallol, Enric I. Canela, Antoni Cortés, Vicent Casadó

**Affiliations:** ^1^Department of Biochemistry and Molecular Biomedicine, Faculty of Biology, University of Barcelona, Barcelona, Spain; ^2^Institute of Biomedicine of the University of Barcelona (IBUB), Barcelona, Spain; ^3^Centro de Investigación Biomédica en Red sobre Enfermedades Neurodegenerativas, Madrid, Spain

**Keywords:** adenosine deaminase, adenosine A_2A_ receptor, bioluminescence resonance energy transfer, CD26, dipeptidyl peptidase IV, moonlighting protein, protein–protein interaction

## Abstract

Adenosine is an endogenous purine nucleoside that acts in all living systems as a homeostatic network regulator through many pathways, which are adenosine receptor (AR)-dependent and -independent. From a metabolic point of view, adenosine deaminase (ADA) is an essential protein in the regulation of the total intracellular and extracellular adenosine in a tissue. In addition to its cytosolic localization, ADA is also expressed as an ecto-enzyme on the surface of different cells. Dipeptidyl peptidase IV (CD26) and some ARs act as binding proteins for extracellular ADA in humans. Since CD26 and ARs interact with ADA at opposite sites, we have investigated if ADA can function as a cell-to-cell communication molecule by bridging the anchoring molecules CD26 and A_2A_R present on the surfaces of the interacting cells. By combining site-directed mutagenesis of ADA amino acids involved in binding to A_2A_R and a modification of the bioluminescence resonance energy transfer (BRET) technique that allows detection of interactions between two proteins expressed in different cell populations with low steric hindrance (NanoBRET), we show direct evidence of the specific formation of trimeric complexes CD26-ADA-A_2A_R involving two cells. By dynamic mass redistribution assays and ligand binding experiments, we also demonstrate that A_2A_R-NanoLuc fusion proteins are functional. The existence of this ternary complex is in good agreement with the hypothesis that ADA could bridge T-cells (expressing CD26) and dendritic cells (expressing A_2A_R). This is a new metabolic function for ecto-ADA that, being a single chain protein, it has been considered as an example of moonlighting protein, because it performs more than one functional role (as a catalyst, a costimulator, an allosteric modulator and a cell-to-cell connector) without partitioning these functions in different subunits.

## Introduction

Many proteins interact with other proteins or are organized into macromolecular complexes, in which multiple components work together to perform different cellular processes (Petschnigg et al., 2012). Transient protein-protein interactions are composed of relatively weak interactions and they perform essential functional roles in biological systems, notably in regulating the dynamic of biological networks (Maleki et al., 2013; Tsuji et al., 2015; Cigler et al., 2017). Investigation of protein–protein interactions of the membrane proteins is of special interest, as they have pivotal roles in cellular processes, they are major targets for the development of new therapeutics and they are often directly linked to human diseases (Petschnigg et al., 2012; Bourgaux and Couvreur, 2014; Jazayeri et al., 2015; Yin and Flynn, 2016; Saraon et al., 2017; Shrivastava et al., 2017). An example of these interactions is that established by the enzyme adenosine deaminase with different proteins (Cortés et al., 2015). Fluorescence resonance energy transfer (FRET) and bioluminescence resonance energy transfer (BRET) are biophysical techniques widely used to analyze direct protein–protein interactions that take place in living cells as well as conformational changes within proteins or molecular complexes (Ciruela, 2008; Kimura et al., 2010; Kim et al., 2011; Lohse et al., 2012; Deriziotis et al., 2014; Brown et al., 2015; Mo and Fu, 2016; Corbel et al., 2017).

Adenosine deaminase (ADA, EC 3.5.4.4) catalyzes the irreversible deamination of adenosine or 2′-deoxyadenosine to inosine or 2-deoxyinosine, respectively. In humans, there are two different enzymes, which are designated ADA1, from here ADA, and ADA2. ADA is a monomeric enzyme that plays a central role in purine metabolism (He et al., 2015; Kutryb-Zajac et al., 2016). Interest in ADA function increased after the discovery that about 15% of inherited immunodeficiencies are caused by mutations in the ADA gene that lead to a loss of function of this protein (Buckley, 2004). This set of disorders is known as severe combined immunodeficiency (SCID), characterized by dysfunction of the T, B, and natural killers (NK) cells and severe lymphopenia. The absence of ADA activity causes lymph-toxic deoxyadenosine triphosphate accumulation that results in apoptosis in immature thymocytes (Buckley, 2004; Shaw et al., 2017; Turel et al., 2017). Different crystal structures of ADA have been obtained, containing a tightly bound Zn^2+^ that is essential for the stability and the catalytic function of the native protein (Wilson et al., 1991; Cooper et al., 1997; Wang and Quiocho, 1998; Kinoshita et al., 2005; Niu et al., 2010; Khare et al., 2012; Bottari et al., 2014; Grosskopf et al., 2017). ADA is a cytosolic enzyme localized in many human tissues, being the lymphoid system (lymph nodes, spleen and thymus) where the highest levels are found (Beyazit et al., 2012; Maiuolo et al., 2016). In the case of ADA2, Zavialov and Engström (2005) showed that the abundance of this enzyme in human tissues is low and that the gene encoding ADA2 is part of a new family of adenosine deaminase growth factors. Likewise, the same authors reported the structure of ADA2, revealing striking differences with ADA both in their global structures and in the arrangement of their catalytic centers (Zavialov et al., 2010).

It has been demonstrated that ADA can also be expressed as an ecto-enzyme on the surface of several cell types, such us lymphocytes (Martín et al., 1995; Blackburn and Kellems, 2005), erythrocytes (Da Silva et al., 2013), dendritic cells (Pacheco et al., 2005; Desrosiers et al., 2007; Casanova et al., 2012), endothelial and epithelial cells (Ginés et al., 2002; Eltzschig et al., 2006), fibroblasts (Torvinen et al., 2002), platelets (Souza Vdo et al., 2012) and neurons (Ruiz et al., 2000; Hawryluk et al., 2012). Up to now, dipeptidyl peptidase IV (DPPIV, EC3.4.14.5, also known as CD26) and some adenosine receptors (ARs), as A_1_R, A_2A_R, and A_2B_R, serve as binding proteins for extracellular ADA in humans (Beckenkamp et al., 2016; Arin et al., 2017). The cluster differentiation antigen CD26, is a ubiquitously expressed multifunctional cell surface serine protease that cleaves dipeptides from the N-terminal end of oligopeptides and smaller peptides with either L-Ala or L-Pro in the penultimate position (Gorrell, 2005; Zhong et al., 2015a; Klemann et al., 2016; Mortier et al., 2016).

Human CD26 is a homodimeric integral membrane type II glycoprotein which is anchored through its signal peptide. The large C-terminal of the extracellular component of CD26 contains an α/β-hydrolase domain and an eight-blade β-propeller domain, that is open and consists of two subdomains responsible for the glycosylation-rich and cysteine-rich regions, respectively. ADA, caveolin-1 and many monoclonal anti-CD26 antibodies bind to the glycosylation-rich domain, while plasminogen, fibronectin, collagen and streptokinase bind to the cysteine-rich region (Klemann et al., 2016). The catalytic region of CD26 is responsible for the enzymatic activity on its natural substrates, including incretins, such as glucagon-like peptide-1 and glucose-dependent insulinotropic peptide, neuropeptides, chemokines, and a few growth factors and cytokines leading to their inactivation and/or degradation (Larrinaga et al., 2015; Zhong et al., 2015b; Mortier et al., 2016). Moreover, a soluble monomeric form of CD26 has been reported in plasma and other body fluids, which enhances the effect of stimulant agents on T-cell proliferation independently of both the enzymatic activity and the ADA-binding ability of CD26 (Yu et al., 2011; Zhong et al., 2015a).

CD26 has been implicated in a variety of pathologies, including rheumatoid arthritis, autoimmune diseases, HIV infection and different types of cancers and CNS tumors (Yu et al., 2006; Songok et al., 2010; Havre et al., 2013; Cordero et al., 2015; Larrinaga et al., 2015; Klemann et al., 2016; Mortier et al., 2016; Beckers et al., 2017; Lee et al., 2017). CD26 has a number of non-enzymatic functions via interactions with several proteins, for instance, ADA, caveolin-1, streptokinase, tyrosine phosphatase, collagen, fibronectin, CD45, chemokine receptor CXCR4, plasminogen type 2, the HIV gp120 protein and the human coronavirus MERS-CoV spike protein (Lambeir et al., 2003; Havre et al., 2008; Fan et al., 2012; Lu et al., 2013; Zhong et al., 2015a; Baerts et al., 2015; Kanno et al., 2016; Xin et al., 2017). As an adhesion molecule, CD26 could facilitate adhesion, migration and metastasis of tumor cells by binding to the extracellular matrix proteins fibronectin and collagen (see Mortier et al., 2016).

The second type of ADA anchoring proteins on the cell surface are the A_1_R (Ciruela et al., 1996; Saura et al., 1996; Sun et al., 2005; Gracia et al., 2008, 2013b), the A_2A_R (Gracia et al., 2011, 2013a) and the A_2B_R (Herrera et al., 2001; Antonioli et al., 2014; Arin et al., 2015, 2017), which are members of the family A of G-protein coupled receptors (GPCRs) (Fredholm et al., 2011). A_1_R is coupled to G_i/o_ proteins, while A_2A_R and A_2B_R are coupled to G_s/olf_ proteins. A_1_Rs inhibit adenylyl cyclase activity through the activation of a G-protein that is sensitive to pertussis toxin, therefore reducing the intracellular levels of cyclic AMP. In contrast, A_2A_R and A_2B_R have a stimulatory effect on adenylyl cyclase activity increasing cyclic AMP levels, with the consequent PKA activation and CREB phosphorylation. The activation of A_2A_R can also activate protein kinase C (PKC), through cyclic AMP-dependent and -independent mechanisms (Jacobson and Gao, 2006; Sheth et al., 2014; Cortés et al., 2015; Leiva et al., 2017). A_1_R and A_2A_R are high affinity receptors with activity in the low to middle nanomolar range whereas A_2B_R has a substantially lower affinity for adenosine (micromolar) (Borea et al., 2015). All ARs are widely expressed and are involved in multiple biological functions, both in physiological and pathological conditions (Chen et al., 2013), including sleep regulation (Lazarus et al., 2017), cardioprotection (Gile and Eckle, 2016), renal function (Welch, 2015), lipolysis (Leiva et al., 2017), immune function (Cekic and Linden, 2016), angiogenesis (Du et al., 2015), as well as ischemia-reperfusion injury (Sharma et al., 2016), inflammation (Cronstein and Sitkovsky, 2017) and neurodegenerative disorders (Huang et al., 2017; Stockwell et al., 2017).

Since CD26 and ARs interact with ADA at opposite sites (Weihofen et al., 2004; Fan et al., 2012; Gracia et al., 2013a), in the present paper we have investigated if ADA could function as a cell-to-cell communication molecule by bridging the anchoring molecules CD26 and A_2A_R present on the surfaces of the interacting cells. We have used a modification of the BRET technique that allows detection of interactions between two proteins expressed in different cell populations with low steric hindrance (NanoBRET) (Machleidt et al., 2015; Mo and Fu, 2016). The results obtained confirm that the cloned A_2A_R-NanoLuc and CD26-YFP constructs express correctly in HEK cells and can form with ecto-ADA oligomeric complexes which can be of metabolic relevance *in vivo*.

## Materials and Methods

### Expression Vectors and Fusion Proteins

Human cDNAs for A_2A_R, NMDAR1A or CD26 protein, cloned into pcDNA3.1, were amplified without their stop codons using sense and antisense primers harboring: EcoRI and KpnI sites to clone A_2A_R in pRluc-N1 vector (p*Rluc*-N1; PerkinElmer, Wellesley, MA, United States), KpnI and BamHI sites to clone A_2A_R or EcoRI and NotI to clone NMDAR1A in Nluc vector (*NanoLuc* Promega, Madison, WI, United States) and EcoRI and KpnI to clone CD26 or HindIII and BamHI to clone NMDAR1A in pEYFP-N1 vector (enhanced yellow variant of GFP; Clontech, Heidelberg, Germany). Amplified fragments were subcloned to be in-frame with restriction sites of pRluc-N1, Nluc or pEYFP-N1 vectors to provide plasmids that express proteins fused to YFP on the C-terminal end (CD26-YFP) or on the N-terminal end (NMDAR1A-YFP) or protein fused to Rluc on the C-terminal end (A_2A_R-Rluc) or Nluc on the N-terminal end (NMDAR1A-Nluc, A_2A_R-Nluc) with and without spacer (GTAGTGCCA). It was observed that all fusion proteins showed a similar membrane distribution as naïve receptors, and fusion of bioluminescent protein to receptor did not modify receptor function as determined by ERK assays. Plasmid pZC11-containing TAC-promoted wild-type human ADA or Leu58Ala or Leu62Ala ADA mutants cDNA were used as previously indicated (Gracia et al., 2013a).

### Antibodies and Purified Proteins

Human-specific monoclonal antibody (mAb) against CD26, TA5.9-CC1-4C8 directed against the ADA-binding epitope on CD26 was previously characterized (Blanco et al., 2000; Pacheco et al., 2005; Martinez-Navio et al., 2009; Casanova et al., 2012). Albumin was purchased from Sigma–Aldrich (St. Louis, MI, United States). Bovine ADA was purchased from Roche (Basel, Switzerland).

### Bacterial Strains and Vector

*Escherichia coli* SΦ3834, a multiple auxotroph (rpsL, Dadduid- man, metB, guaA, uraA: Tn 10) with a deletion of add (bacterial ADA gene), and plasmid pZC11-containing TAC-promoted wild-type human ADA cDNA (Chang et al., 1991) and Leu58Ala and Leu62Ala ADA mutants cDNA were used (Gracia et al., 2013a). Overnight cultures of pZC11-hADA transformants of SΦ3834 were inoculated into the appropriate volume of Luria-Bertani (LB) medium supplemented with carbenicillin (200 μg/ml) and tetracycline (18.75 μg/ml) (Sigma–Aldrich). Cells were grown with shaking at 37°C until an A_600 nm_ = 1.0 and then were harvested and frozen at -80°C (Richard et al., 2002; Gracia et al., 2008).

### Partial Purification of ADA

Recombinant wild-type and ADA mutants were partially purified from 500 ml cultures of *E. coli* SΦ3834 cells, and transformed with the plasmid pZC11 containing the cDNA of ADA, according to Gracia et al. (2013a). Briefly, cell pellets were resuspended at 4°C in 5 ml of lysis buffer. The suspensión was cooled on ice, and sonicated for 24 s × 20 s at 15% intensity in a sonifier (Branson Ultrasonics Corp., Danbury, CT, United States). The homogenate was centrifuged at 105,000 × *g* for 60 min, and protamine sulfate (Sigma–Aldrich) was slowly added up to a final concentration of 2 mg/ml. After 60 min of constant stirring, the suspension was again centrifuged, and the supernatant was desalted with a PD10 (GE Healthcare) gel filtration column, preequilibrated with 50 mM, pH 7.4, Tris-HCl buffer, and stored at 4°C for their immediate use.

### Enzyme Activity and Kinetic Parameters of ADA

Adenosine deaminase activity was determined at 25°C with 0.1 mM adenosine as substrate in 50 mM Tris-HCl buffer, pH 7.4, as previously reported (Gracia et al., 2013a). The decrease in the absorbance at 265 nm (Δ𝜀 = 7800 M^-1^ cm^-1^) was monitored in an Ultrospec 3300 pro spectrophotometer (Biochrom Ltd., Cambridge, United Kingdom) with 1-ml cuvettes. One unit (U) of ADA activity is defined as the amount of enzyme required to hydrolyze 1 μmol of adenosine per minute in the assay conditions. Steady-state kinetic measurements were performed in 50 mM Tris-HCl buffer (pH 7.4) using a concentration range of adenosine from 10 μM to 1 mM and a constant enzyme concentration. Inhibition studies were carried out by monitoring the hydrolysis rates of adenosine in the presence of constant concentrations of purine riboside (from 5 μM to 0.5 mM; Sigma–Aldrich) (Gracia et al., 2013a). In all cases, a minimum of four replicates for each single experimental point were performed. Kinetic parameters were obtained by fitting the data to the appropriate rate equations, using a non-linear regression software (Grafit, Erithacus Software, Surrey, United Kingdom).

### Cell Culture and Transient Transfection

Human embryonic kidney (HEK-293T) cells obtained from ATCC were grown in Dulbecco’s modified Eagle’s medium (DMEM) (Gibco) supplemented with 100 μg/ml sodium pyruvate, 2 mM L-glutamine, 100 U/ml penicillin/streptomycin, essential medium non-essential amino acids solution (1/100) and 5% (v/v) heat inactivated fetal bovine serum (all from Invitrogen, Paisley, United Kingdom) and were maintained at 37°C in an atmosphere with 5% CO_2_. Cells growing in 6-well dishes were transiently transfected with the corresponding protein cDNA by the polyethylenimine method (Sigma–Aldrich). Cells were incubated with the corresponding cDNA together with polyethylenimine (5.47 mM in nitrogen residues) and 150 mM NaCl in a serum-starved medium. After 4 h, the medium was renewed and 48 h after transfection, cells were washed twice in quick succession in HBSS [containing 137 NaCl, 5 KCl, 1.26 CaCl_2_, 0.4 MgSO_4_, 0.5 MgCl_2_, 0.34 Na_2_HPO_4_, 0.44 KH_2_PO_4_, 10 HEPES, pH 7.4, in mM], supplemented with 10 mM glucose, detached, and resuspended in the same buffer. Protein concentration was determined using the Bradford assay (Bio-Rad, Munich, Germany), in order to control cell number.

### Immunodetection Assays

Cells were fixed in 4% paraformaldehyde for 15 min and washed with PBS containing 20 mM glycine (buffer A) to quench the aldehyde groups. Then, cells were permeabilized with buffer A containing 0.2% Triton X-100 for 5 min, and treated with 1% of BSA in PBS. After 1 h at room temperature, cells were labeled with the primary mouse anti-A_2A_R antibody (1:200; Millipore, Darmstadt, Germany; cat #05-717) for 1 h to detect A_2A_R–Nluc, washed, and stained with the secondary goat anti-mouse Alexa Fluor 488 (1:300; Invitrogen, Paisley, United Kingdom; cat #A-11001). The specificity of this antibody for immunocytofluorescence studies has been previously reported by Moreno et al. (2017a). CD26-YFP fused to YFP protein was detected by its fluorescence properties. The samples were rinsed several times and mounted with 30% Mowiol (Calbiochem) as reported by Moreno et al. (2017b). Samples were observed in a Leica SP2 confocal microscope.

### ERK Phosphorylation Assay

HEK-293T cells expressing A_2A_R were cultured in serum-free medium for 16 h before the addition of any agent. Cells were treated at 25°C with 100 nM CGS 21680 (Sigma–Aldrich) for 10 min and rinsed with ice-cold PBS. Cells were lysed by ice-cold lysis buffer (1% Triton X-100, 150 mM NaCl, 50 mM Tris-HCl pH 7.4, 50 mM NaF, 45 mM β-glycerophosphate, 20 mM phenyl-arsine oxide, 0.4 mM NaVO_4_ and protease inhibitor cocktail). The cellular debris was removed by centrifugation at 13,000 × *g* for 5 min at 4°C and the protein was quantified by the bicinchoninic acid method using bovine serum albumin as standard. The level of ERK 1/2 phosphorylation was determined in equivalent amounts of protein (10 μg) separated by electrophoresis on 7.5% SDS polyacrylamide gel and transferred onto PVDF-FL membranes, according to Gracia et al. (2013b). Odyssey blocking buffer (LI-COR Biosciences, Lincoln, NE, United States) was used for 90 min. Membranes were then probed with a mixture of a mouse anti-phospho-ERK 1/2 antibody (1:2500, Sigma) and rabbit anti-ERK 1/2 antibody (1:40,000, Sigma) for 2–3 h. Bands were visualized after 1 h incubation with a mixture of IRDye 800 (anti-mouse) antibody (1:10,000, Sigma) and IRDye 680 (anti-rabbit) antibody (1:10,000, Sigma) and scanned by the Odyssey infrared scanner (LICOR Biosciences, Lincoln, NE, United States). Bands densities were quantified and the level of phosphorylated ERK 1/2 isoforms was normalized according to the total ERK 1/2 protein bands (Gracia et al., 2013b).

### Dynamic Mass Redistribution (DMR) Assays

The cell signaling signature was determined using an EnSpire^®^ Multimode Plate Reader (PerkinElmer, Waltham, MA, United States) by a label-free technology. Refractive waveguide grating optical biosensors, integrated into 384-well microplates, allow the measurement of changes in local optical density in a detecting zone up to 150 nm above the surface of the sensor. Cellular mass movements induced after activation of the receptors are detected by illuminating the lower part of the biosensor with polychromatic light, determining changes in wavelength of the reflected monochromatic light, which are a sensitive function of the refractive index. The magnitude of this wavelength shift (in picometers) is directly proportional to the amount of DMR (Viñals et al., 2015). The assay was carried out according to Viñals et al. (2015); briefly, 24 h before the assay, cells were seeded in 384-well sensor microplates at a density of 10,000–12,000 cells per well, with 30 μl growth medium and were cultured for 24 h (37°C, 5% CO_2_) until reaching 70–80% confluent monolayers. For the assay, cells were washed twice with assay buffer (HBSS with 20 mM HEPES, pH 7.15) and incubated for 2 h in 30 μl per well of assay-buffer with 0.1% DMSO in the reader at 24°C. Then, the sensor plate was scanned and a baseline optical signature was recorded before adding 10 μl of receptor agonist dissolved in assay buffer containing 0.1% DMSO. DMR responses were monitored for at least 5,000 s and kinetic results were analyzed using the EnSpire Workstation software v 4.10.

### Determination of cAMP Concentration

cAMP production was determined according to Viñals et al. (2015), using a homogeneous time-resolved fluorescence energy transfer (HTRF) assay with the Lance Ultra cAMP kit (PerkinElmer, Waltham). We first established the optimal cell density to obtain an appropriate TR-FRET signal within the dynamic range of a standard cAMP curve. This was done by measuring the basal and activated TR-FRET signal using different cell densities. Cells (1,000 cells/well) growing at 25°C in white ProxiPlate 384-well microplates (PerkinElmer) with a medium containing 50 μM zardaverine were stimulated with 100 nM CGS 21680 (Sigma–Aldrich) for 10 min or treated with vehicle. Fluorescence at 665 nm was analyzed on a PHERAstar Flagship microplate reader equipped with an HTRF optical module (BMG Lab technologies, Offenburg, Germany).

### Nano Bioluminescence Resonance Energy Transfer (NanoBRET) between Two Proteins Expressed in Two Different Cell Populations

HEK-293T cells were transiently transfected with the corresponding donor o acceptor, 48 h after transfection, cells expressing the donor were mixed with HEK-293 cells expressing the acceptor. Cells were incubated 10 min with HBSS without shaking in the presence or in the absence of wild-type ADA, albumin, TA5.9-CC1-4C8 antibody or Leu58Ala and Leu62Ala ADA mutants. Protein-YFP expression was quantified by distributing cells (20 μg protein, around 4000 cells/well) in 96-well microplates (black plates with a transparent bottom) and fluorescence was read at 400 nm in a Fluo Star Optima Fluorimeter (BMG Labtechnologies), equipped with a high-energy xenon flash lamp, using a 10 nm bandwidth excitation filter. Protein fluorescence expression was determined as fluorescence of the sample minus the fluorescence of cells expressing Nluc alone. For NanoBRET measurements, the equivalent of 20 μg of mixed cells were distributed in 96-well microplates (Corning 3600, white plates; Sigma) and 5 μM of coelenterazine H (Molecular Probes, Eugene, OR) was added. After 1 min, readings were collected using a Mithras LB 940 late reader (Berthold Technologies) that allows the integration of the signals detected in the short-wavelength filter at 440–500 nm and the long-wavelength filter at 510–590 nm. To quantify receptor-Nluc expression, bioluminescence readings were also performed after 10 min of adding 5 μM of coelenterazine H. Fluorescence and bioluminescence of each sample were measured before every experiment to confirm similar donor expressions (approximately 120,000 bioluminescence units per 20 μg of protein) while acceptor expression (25,000 fluorescence units per 20 μg of protein). The net BRET was defined as [(long-wavelength emission)/(short-wavelength emission)]-Cf where Cf corresponds to [(long-wavelength emission)/(short-wavelength emission)] for the receptor-Nluc expressed alone in the same experiment. BRET is expressed as milliBRET units (mBU).

### Cell Membranes Preparation and Radioligand Binding Experiments

Human embryonic kidney cells transfected with A_2A_R non-fused or fused to Nluc-spacer were disrupted with a Polytron homogenizer (PTA 20 TS rotor, setting 3; Kinematica, Basel, Switzerland) for three 5 s-periods in 10 volumes of 50 mM Tris–HCl buffer, pH 7.4 containing a proteinase inhibitor cocktail. Cell debris was removed by centrifugation at 1,000 × *g* (5 min, 4°C) and membranes were obtained by centrifugation at 105,000 × *g* (40 min, 4°C). The pellet was resuspended and re-centrifuged under the same conditions and was stored at -80°C. Membranes were washed once more as described above and resuspended in 50 mM Tris–HCl buffer, pH 7.4 containing 10 mM MgCl_2_. Membrane protein was quantified by the bicinchoninic acid method (Pierce Chemical Co., Rockford, IL, United States) using bovine serum albumin dilutions as standard. For A_2A_R competition-binding assays, membrane suspensions (0.2 mg of protein/ml) were incubated for 2 h at 25°C with a constant free concentration of 2.2 nM of the A_2A_R antagonist [^3^H]ZM 241385 (50 Ci/mmol; American Radiolabeled Chemicals, St. Louis, MO, United States) and increasing concentrations of unlabelled ZM 241385 (Tocris, Ellisville, MO, United States), in the absence or in the presence of bovine ADA. In dose-response curves of ADA, membranes were also incubated with 2.2 nM [^3^H]ZM 241385 and with increasing concentrations of bovine ADA. In all cases, non-specific binding was determined in the presence of 10 μM of unlabelled ZM 241385. Free and membrane-bound ligands were separated by rapid filtration of 500 μl aliquots in a cell harvester (Brandel, Gaithersburg, MD, United States) through Whatman GF/C filters embedded in 0.3% polyethylenimine that were subsequently washed for 5 s with 5 ml of ice-cold 50 mM Tris-HCl buffer. The filters were incubated overnight with 10 ml of Ultima Gold MV scintillation cocktail (PerkinElmer) at room temperature and radioactivity counts were determined using a Tri-Carb 2800 TR scintillation counter (PerkinElmer) with a mean efficiency of 62%.

Data were analyzed according to the ‘two-state dimer model.’ This model assumes GPCR dimers as a main functional unit and provides a more robust analysis of parameters obtained from saturation and competition experiments with orthosteric ligands, as compared with the commonly used ‘two-independent-site model’ (Casadó et al., 2007, 2009a,b). In competition experiments the model analyzes the interactions of the radioligand with a competing ligand and it provides the affinity of the competing ligand for the first protomer in the unoccupied dimer (*K*_DB1_) and the affinity of the competing ligand for the second protomer when the first protomer is already occupied by the competing ligand (*K*_DB2_). Radioligand competition curves were analyzed by non-linear regression using the commercial Grafit software (Erithacus Software, Surrey, United Kingdom). To calculate the macroscopic equilibrium dissociation constants from competition experiments, the following general equation 1 must be applied:

(1) $$A_{\mathrm{bound}}=\frac{(K_{\mathrm{DA}2}\;A\;+\;2A^{2}\;+\;\frac{K_{\mathrm{DA}2}A\;B}{K_{\mathrm{DAB}}})\;R_{T}}{K_{\mathrm{DA}1}\;K_{\mathrm{DA}2}\;+\;K_{\mathrm{DA}2}\;A\;+\;A^{2}\;+\;\frac{K_{\mathrm{DA}2}\;A\;B}{K_{\mathrm{DAB}}}\;+\;\frac{K_{\mathrm{DA}1}\;K_{\mathrm{DA}2}\;B}{K_{\mathrm{DB}1}}\;+\;\frac{K_{\mathrm{DA}1}\;K_{\mathrm{DA}2}\;B^{2}}{K_{\mathrm{DB}1}\;K_{\mathrm{DB}2}}\;}$$

where *A* represents the radioligand concentration, *B* the assayed competing compound concentration, *K*_Dn_ the equilibrium dissociation constant of the first or second binding of *A* or *B* to the dimer, and *K*_DAB_ the hybrid allosteric modulation between *A* and *B*. For *A* and *B* being the same non-cooperative ligand, the equation 1 can be simplified to equation 2 (Gracia et al., 2013b):

(2) $$A_{\mathrm{bound}}=\frac{(4K_{\mathrm{DA}1}A+2A^{2}+AB)R_{T}}{4K_{\mathrm{DA}1}^{2}+4K_{\mathrm{DA}1}A+A^{2}+AB+4K_{\mathrm{DA}1}B+B^{2}}$$

## Results

### Expression and Characterization of A_2A_R and CD26 Fusion Proteins

The aim of this paper is to investigate the ADA-mediated molecular interaction between A_2A_R expressed in the membrane of one cell and CD26 expressed in the membrane of another cell by the NanoBioluminiscence Resonance Energy Transfer (NanoBRET) technique. This biophysical technique has been extensively validated to analyze direct protein–protein interactions occurring in living cells. For any approximation based in transfer of energy it is necessary a donor, a protein fused to the enzyme NanoLuc (Nluc) and an acceptor, a protein fused to the fluorescent protein YFP. Here we used A_2A_R fused to Nluc (A_2A_R-Nluc) or to Nluc with a spacer (A_2A_R-Nluc-spacer) and CD26 fused to YFP (CD26-YFP). To detect NanoBRET with a donor in one cell and an acceptor in another cell is necessary that fusion proteins are placed at the extracellular space. One of the main drawbacks in this case is that the bioluminescent protein fused at the N-terminus of A_2A_R could disturb the expression and/or the ligand binding to the receptor giving a non-functional receptor or block the unknown ADA binding site of the receptor. We have used the enzyme Nluc to improve protein translocation of fused complexes and to reduce the volume of the bioluminescent enzyme fused to A_2A_R. Moreover, the “combination of greater light intensity with improved spectral resolution results in substantially increased detection sensitivity and dynamic range over current BRET technologies” (Machleidt et al., 2015). In the case of CD26, the binding domain of ADA has been described previously (Weihofen et al., 2004), and given that it is located in a middle area of the extracellular domain, the binding of ADA to CD26 should not be hindered by the binding of the YFP protein at the C-terminal end of CD26. Taken this into account, we first characterized the fusion proteins.

**FIGURE 1:**
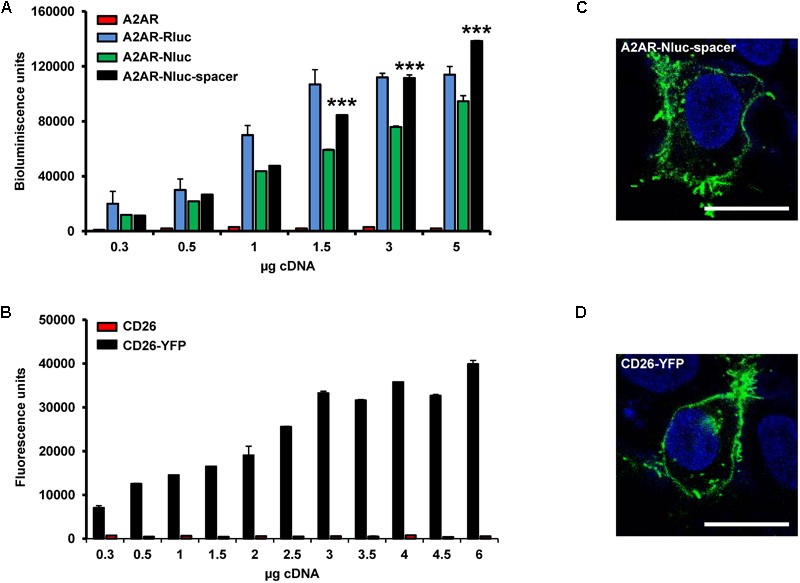
Expression of A_2A_R and CD26 fusion proteins. **(A)** Bioluminescence assays were performed in HEK-293T cells transfected with increasing concentrations of fusion protein cDNA corresponding to A_2A_R (red), A_2A_R-Rluc fused on the C-terminal end (blue), A_2A_R-Nluc fused on the N-terminal (green) or A_2A_R-Nluc-spacer fused on the N-terminal end (black) on the N-terminal end. Results are given in relative bioluminescence units by subtracting the value of non-transfected cells and represent mean ± SEM (*n* = 6). Statistical significance was calculated by one-way ANOVA followed by a Bonferroni multiple comparison *post hoc* test; ^∗∗∗^*p* < 0.001 against A_2A_R-Nluc. **(B)** Fluorescence assays were performed in HEK-293T cells transfected with increasing concentrations of CD26 (red) or CD26-YFP fused on the C-terminal end (black). Results are given in relative fluorescence units by subtracting the value of non-transfected cells and represent mean ± SEM (*n* = 10). **(C,D)** Confocal microscopy images from immunofluorescence experiments using HEK-293T cells transfected with 0.75 μg of A_2A_R-Nluc-spacer fused on the N-terminal end **(C)** or 1 μg of CD26-YFP fused on the C-terminal end **(D)** are shown. Immunocytofluorescence experiments were carried out with anti-A_2A_R primary antibody (1:100; Millipore) and goat anti-mouse Alexa Fluor 488 (1:300; Invitrogen) as secondary antibody. YFP-fused proteins were identified by their own fluorescence. A_2A_R-Nluc-spacer and CD26-YFP are labeled in green. Nuclei are colored in blue by DAPI staining. Scale bar: 20 μM.

HEK-293T cells were transfected with increasing concentrations of cDNA for A_2A_R-Nluc or A_2A_R-Nluc-spacer or with increasing concentrations of cDNA for non-fused A_2A_R as negative control or A_2A_R-Rluc fused at the C-terminal domain as positive control and bioluminescence was measured (**Figure 1A**). All fusion proteins were expressed and A_2A_R-Nluc-spacer was significantly better expressed compared to A_2A_R-Nluc, reaching expression values similar to the ones obtained for common Rluc fused at the C-terminus domain of A_2A_R (**Figure 1A**). HEK-293T cells were also transfected with increasing concentrations of cDNA for CD26-YFP or non-fused CD26 as negative control and fluorescence was measured (**Figure 1B**). We observed that the fusion protein was expressed. Next we tested by confocal microscopy that A_2A_R-Nluc-spacer (**Figure 1C**) and CD26-YFP (**Figure 1D**) showed a plasma membrane distribution as expected.

**FIGURE 2:**
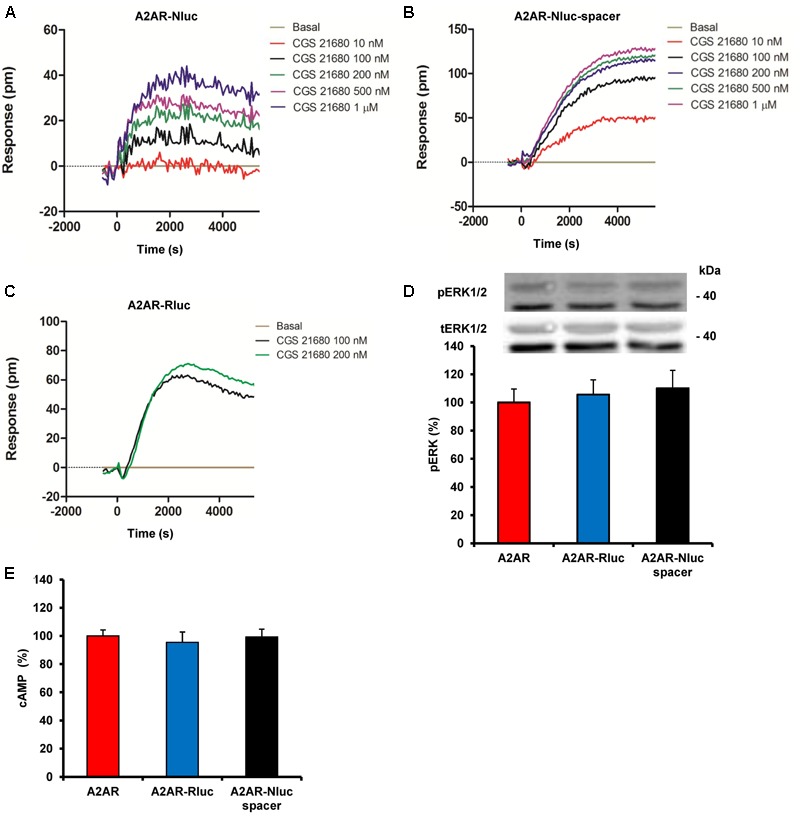
Functional characterization of A_2A_R fusion proteins. **(A–C)** DMR assays were performed in HEK-293T cells transfected with cDNA (1.5 μg) corresponding to A_2A_R-Nluc **(A)** or A_2A_R-Nluc-spacer **(B)** both fused on the N-terminal end or A_2A_R-Rluc fused on the C-terminal end **(C)**. Cells were stimulated with vehicle (basal) or with increasing concentrations of the A_2A_R agonist CGS 21680. The resulting shifts of reflected light wavelength (pm) were monitored over time. Each panel is a representative experiment of *n* = 3 different experiments. Each curve is the mean of a representative optical trace experiment carried out in quadruplicates. **(D,E)** ERK1/2 phosphorylation **(D)** and cAMP production **(E)** were determined in cells transfected with the cDNA (1.5 μg) corresponding to A_2A_R, A_2A_R-Rluc fused on the C-terminal end or A_2A_R-Nluc-spacer fused on the N-terminal end. Cells were stimulated with 100 nM CGS 21680 for 10 min. Results are given as percentage respect cells expressing only A_2A_R. Values are expressed as means ± SEM (*n* = 4). **(D)** A representative western blot is shown at the top of the panel and in **(E)** 100% represents 80–100 pmols of cAMP/10^6^ cells.

To evaluate the functional characteristics of the A_2A_R constructs we measured the global cellular response using the DMR label-free assay. This technique detects agonist-induced changes in light diffraction in the bottom 150 nm of a cell monolayer (see section “Materials and Methods”). HEK-293T cells were transfected with cDNA corresponding to A_2A_R-Nluc (**Figure 2A**), A_2A_R-Nluc-spacer (**Figure 2B**), or A_2A_R-Rluc (**Figure 2C**). Cells were stimulated with increasing concentrations of the A_2A_R agonist CGS 21680, and DMR signal was obtained against time. From DMR curves (**Figures 2A–C**) it is observed that A_2A_R-Nluc-spacer gives higher signaling than A_2A_R-Nluc and the signal was similar to the one obtained for common A_2A_R-Rluc construction (**Figures 2A–C**). The lost of functionality of A_2A_R-Nluc respect to the A_2A_R-Rluc indicates that the fused Nluc probably disturbs the agonist binding; thus, introducing the spacer between A_2A_R N-terminal and Nluc, that allows Nluc moving away from the membrane surface, not only favors the fusion protein expression (see **Figure 1A**) but also ligand binding and the corresponding signaling. Moreover, we checked if agonist activation of A_2A_R-Nluc-spacer was able to induce second messengers as naïve receptors. To do this, HEK-293T cells were transfected with cDNA corresponding to A_2A_R-Nluc-spacer or A_2A_R-Rluc or A_2A_R as controls. Cells were stimulated with A_2A_R agonist CGS 21680 (100 nM) and ERK 1/2 phosphorylation and cAMP production were determined. We detected similar extend of ERK 1/2 phosphorylation (**Figure 2D**) and similar cAMP accumulation (**Figure 2E**) in all cells, showing that A_2A_R-Nluc-spacer is fully functional. According to this, the A_2A_R-Nluc-spacer was selected for further studies.

### ADA Binding to A_2A_R Fusion Protein

**FIGURE 3:**
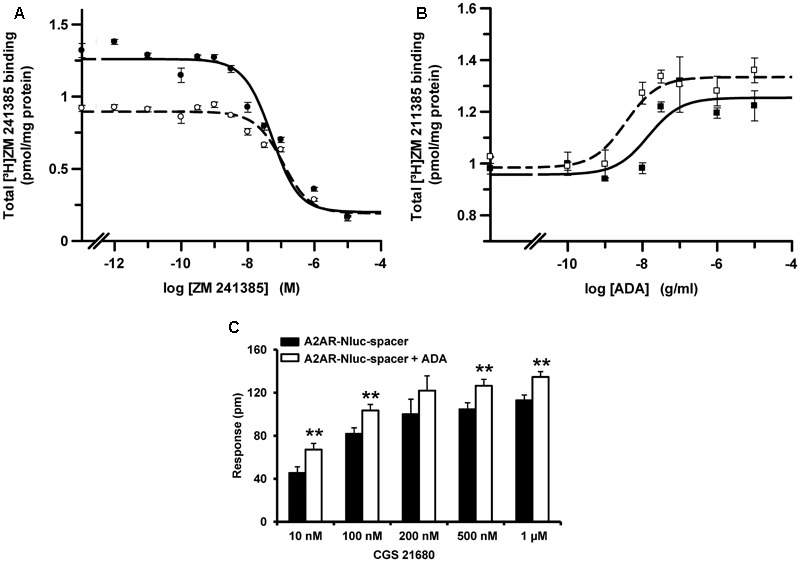
Effect of bovine ADA binding on A_2A_R. **(A)** Competition experiments of 2.2 nM [^3^H]ZM 241385 binding in the presence of increasing concentrations of unlabeled ZM 241385, in the absence (○) or in the presence (●) of 1 μg/ml of ADA were performed using membranes from HEK-293T cells transfected with A_2A_R-Nluc-spacer cDNA (1.5 μg). **(B)** Dose-response effect of ADA on 2.2 nM [^3^H]ZM 241385 binding to membranes from HEK-293T cells transfected with cDNA (1.5 μg) corresponding to A_2A_R-Nluc-spacer (■) or A_2A_R (□). Data are mean ± SD from a representative experiment (*n* = 3) performed in triplicate. **(C)** DMR assays were performed in HEK-293T cells transfected with A_2A_R-Nluc-spacer cDNA (1.5 μg). Cells were stimulated with increasing CGS 21680 concentrations in the presence (white columns) or in the absence (black columns) of ADA (1 μg/ml). Values are mean ± SEM (*n* = 4) and are expressed as shift at 3000 s of reflected light wavelength (pm) over basal obtained from the corresponding DMR curves. Statistical significance was calculated by one-way ANOVA followed by a Bonferroni multiple comparison *post hoc* test; ^∗∗^*p* < 0.01 against samples without ADA.

One characteristic of A_2A_R is their ability to bound extracellular ADA. It has been described that ADA increases the receptor ligand binding affinity and potentiates the receptor functionality (Gracia et al., 2011). Here we analyzed the ability of ADA to bind and modulate A_2A_R-Nluc-spacer fusion protein. We first determined if ADA increases the affinity parameters of antagonist binding to A_2A_R-Nluc-spacer. Ligand binding was analyzed using membranes from HEK-293T cells transfected with cDNA corresponding to A_2A_R-Nluc-spacer. Competition experiments with the A_2A_R antagonist [^3^H]ZM 241385 were performed with increasing concentrations of unlabelled ZM 241385 from 0.001 nM to 10 μM in the absence or in the presence of 1 μg/ml bovine ADA. All curves (**Figure 3A**) are monophasic (*D*_C_ = 0), according to the non-cooperative behavior expected for a A_2A_R ligand binding (Gracia et al., 2011). Moreover, in the presence of ADA, the competition curve of A_2A_R antagonist shifts to the left, indicating an increase in the affinity. The equilibrium binding parameters obtained according to equation (2) (see section “Materials and Methods”) from curves in **Figure 3A** are shown in **Table 1**. When membranes of HEK-293T cells, transfected with cDNA from A_2A_R fused or not to NanoLuc-spacer, were incubated with increasing concentrations of ADA (from 0.1 ng/ml to 10 μg/ml) and with the radiolabeled A_2A_R antagonist, ADA enhanced in a dose-dependent manner the antagonist binding to both A_2A_R non-fused and fused to NanoLuc-spacer (**Figure 3B**). The EC_50_ values obtained with the two proteins are not significantly different (see **Table 1**). These results indicate that ADA can also bind to A_2A_R-Nluc-spacer and significantly increases antagonist affinity. It point out that ADA exerts positive modulation on the antagonist binding to A_2A_R-Nluc-spacer similar to the one obtained for the naïve A_2A_R (**Figure 3B**; and see Gracia et al., 2011, 2013a).

**Table 1:** Effect of adenosine deaminase (ADA) on binding of A_2A_R antagonist [^3^H]ZM 241385.

Binding experiment			ADA	Parameters^a^		
Assay type	Increasing effector	Transfected AR	1 μg/ml	*K*_DA1_^b^	*D*_C_^c^	EC_50_^d^
Competition	ZM 241385	A_2A_R-Nluc-spacer	-	90 ± 20	0	
		A_2A_R-Nluc-spacer	+	30 ± 10^∗∗^	0	
Dose-response	Bovine ADA	A_2A_R-Nluc-spacer				14 ± 8
		A_2A_R				4 ± 2

*Competition curves with ZM 241385 in the absence (-) or the presence (+) of bovine ADA and dose-response curves with bovine ADA were carried out as described in section “Materials and Methods,” using membranes of transfected HEK-293T cells. ^*a*^Values are mean ± SD of three independent experiments performed in triplicate. ^*b*^*K*_*DA1*_ (nM) is the equilibrium dissociation constant of the binding of the radioligand to the first protomer in the dimer. ^*c*^*D*_*C*_ is the dimer cooperativity index for the binding of the radioligand. Note that when *D*_*C*_ = 0 the radioligand is non-cooperative and *K*_*DA2*_ = 4*K*_*DA1*_. ^*d*^EC_*50*_ (ng/ml) is the concentration of ADA providing half-maximal increase in radioligand binding. ^∗∗^*P* < 0.01 against control without ADA; statistical differences were evaluated using Student’s *t*-test.*

To investigate if ADA binding to A_2A_R-Nluc-spacer also increases the receptor functionality, DMR label-free assays were performed in HEK-293T cells transfected with cDNA corresponding to A_2A_R-Nluc-spacer. Cells were not treated or treated with ADA and were stimulated with increasing concentrations of A_2A_R agonist CGS 21680. DMR signal was measured against time. From DMR curves, the response at 3000 s was calculated and plotted as a function of CGS 21680 concentrations used (**Figure 3C**). As in ligand binding experiments, it is observed a significantly increase in the response in the presence of ADA. This indicates that ADA exerts positive modulation on the A_2A_R-Nluc-spacer signaling similar to the one reported for the naïve receptor (Gracia et al., 2011).

### ADA Mediates Cell to Cell Contact by Simultaneous Binding to A_2A_R and CD26

**FIGURE 4:**
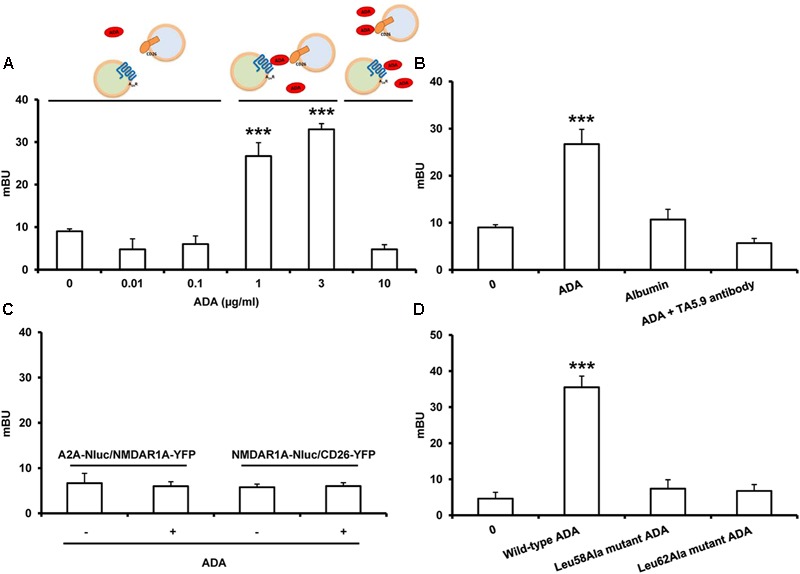
NanoBRET between A_2A_R-Nluc and CD26-YFP expressed in different cells. HEK-293T cells transfected with 1.5 μg of A_2A_R-Nluc-spacer cDNA **(A–D)** or NMDAR1A-Nluc **(C)** were mixed with HEK-293T cells transfected with 2 μg of CD26-YFP cDNA **(A–D)** or 2 μg of NMDAR1A-YFP cDNA **(C)**. Cells were incubated 10 min without shaking with HBSS in the absence or in the presence of increasing concentrations of ADA (from 0.01 to 10 μg/ml) **(A)**, in the presence or in the absence of 1 μg/ml of bovine ADA **(B,C)**, bovine albumin (1 μg/ml) **(B)**, human-specific mAb against CD26, TA5.9-CC1-4C8 (0.3 μg/ml) **(B)** or in the presence or in the absence of human wild-type ADA, Leu58Ala mutant ADA or Leu62Ala mutant ADA, all at 1 μg/ml **(D)**, previously to BRET detection. Both fluorescence and luminescence of each sample were measured before every experiment to confirm similar donor expressions (approximately 120.000 bioluminescence units) and similar acceptor expression (25.000 fluorescence units). BRET is expressed as milliBRET units (mBU = net BRET × 1000) and is means ± SEM of 3–4 different experiments grouped as a function of the amount of BRET acceptor. Statistical significance was calculated by one way ANOVA followed by a Dunnett’s multiple comparison *post hoc* test; ^∗∗∗^*p* < 0.001 compared with the corresponding untreated cells. At the top of the **(A)**, a schematic representation of the effect of different ADA concentrations on the interaction between A_2A_R-Nluc-spacer and CD26-YFP is shown.

To investigate if ADA can induce cell to cell contacts by simultaneous binding to A_2A_R in one and to CD26 in another cell, NanoBRET experiments between cells expressing the NanoBRET donor and cells expressing the NanoBRET acceptor were performed. A_2A_R-Nluc-spacer and CD26-YFP cDNA were transfected separately into different cells. Both cell populations were mixed in the presence and absence of ADA and were allowed to sediment rapidly to facilitate their proximity. If the interaction occurs, the energy transfer between A_2A_R-Nluc-spacer and CD26-YFP could subsequently take place and it could be detected as NanoBRET signal. HEK-293T cells were transfected with increasing concentrations of cDNA corresponding to A_2A_R-Nluc-spacer or CD26-YFP and those samples showing approximately 120.000 bioluminescence units and 25.000 fluorescence units were chosen to perform NanoBRET experiments. Equal number of transfected cells from both types was mixed as well as A_2A_R-Nluc-spacer expressing cells and non-transfected cell or CD26-YFP expressing cells and non-transfected cell as controls and cells were incubated with increasing bovine ADA concentrations, before NanoBRET detection. As shown in **Figure 4A**, we only obtained positive NanoBRET signal in the presence of 1 and 3 μg/ml of ADA, which points out that CD26 and A_2A_R are in close proximity. It is interesting to note that at higher concentrations of ADA (10 μg/ml) the energy transfer decreased. This could be due to the fact that an excess of ADA could saturate the A_2A_R on cells expressing them as well as saturating CD26 protein on the other cells, thereby when both cell types approach, ADA cannot act as a bridge between A_2A_R and CD26, avoiding energy transfer (see schemes in **Figure 4A**, top panels). The results shown in **Figure 4A** show that ADA bridges CD26 and A_2A_R in a narrow range of concentrations, and the optimal ADA concentration required to observe the ternary complex is around 1–3 μg/ml. This is in agreement with the affinity constants of ADA to bind to these two proteins. It has been reported that the affinity of ^125^I-ADA by CD26 is around 18 nM (Gonzalez-Gronow et al., 2004) (equivalent to 0.7 μg/ml), and its affinity by A_1_R is around 230 nM (Saura et al., 1996) (equivalent to 9 μg/ml). These values indicate that first ADA binds to CD26 and then to AR, so that a balance of ADA concentrations occurs between the bars of 0.1 and 10 μg/ml in **Figure 4A** to form a trimeric complex, where ADA has enough concentration to bridge CD26 and AR, but higher concentrations shift the equilibrium toward dimeric ADA-CD26 and ADA-AR complexes.

To determine the specificity of this interaction, equal number of HEK-293T cells transfected with A_2A_R-Nluc-spacer (expressing 120.000 bioluminescence units) were mixed with HEK-293T cells transfected with CD26-YFP (expressing 25.000 fluorescence units) and were incubated with medium (0), with 1 μg/ml bovine ADA, with 1 μg/ml albumin as non-specific protein or with bovine ADA plus 0.3 μg/ml of the human-specific mAb against CD26, TA5.9-CC1-4C8, which is directed against the ADA-binding epitope on CD26 and blocks ADA binding to CD26 (Blanco et al., 2000; Pacheco et al., 2005; Martinez-Navio et al., 2009; Casanova et al., 2012). In these cells, positive NanoBRET signal was only significantly detected in the presence of ADA but not in the presence of albumin or ADA plus TA5.9-CC1-4C8 (**Figure 4B**), showing that ADA specifically mediates A_2A_R-CD26 interaction between different cells. Moreover, when HEK-293T cells transfected with A_2A_R-Nluc-spacer or with NMDAR1A-Nluc (both expressing 120.000 bioluminescence units) were mixed with HEK-293T cells transfected with the metabotropic glutamate receptor subunit NMDAR1A-YFP or with CD26-YFP (both expressing 25.000 fluorescence units), respectively, as negative controls, none NanoBRET signal was detected in the absence or in the presence of ADA (**Figure 4C**) again demonstrating the specificity of the interaction.

**FIGURE 5:**
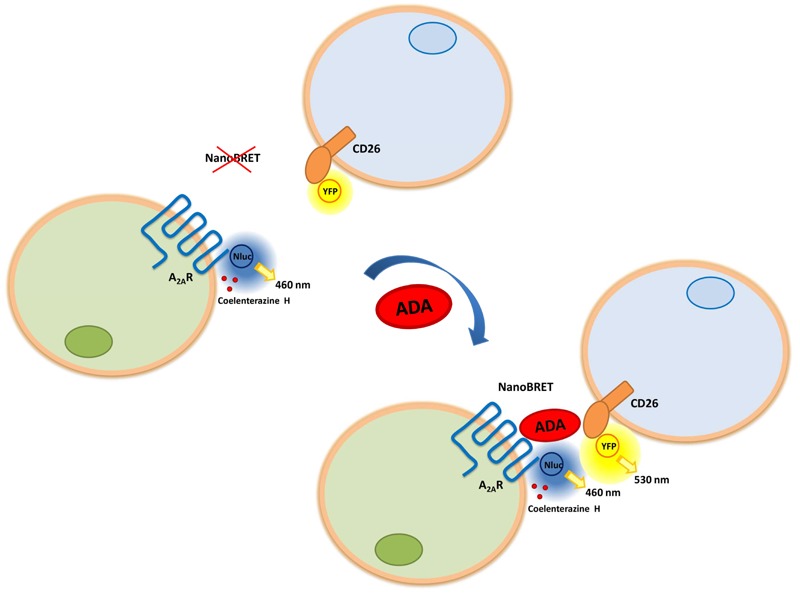
Adenosine deaminase linking A_2A_R and CD26 expressed in different cells. Schematic representation of the effect of ADA on the NanoBRET between A_2A_R-Nluc-spacer (on the N-terminal end) acting as a donor and CD26-YFP (on the C-terminal domain) acting as an acceptor. A_2A_R adenosine receptor (blue), in the absence (left) or in the presence (right) of ADA (red) and CD26 protein (orange), are represented as complexes between cell membranes of different cell types. In the NanoBRET process between A_2A_R-Nluc-spacer expressing cells and CD26-YFP expressing cells, the catalysis of the substrate coelenterazine H emits at 460 nm, allowing YFP excitation and concomitant emission at 530 nm. For simplicity, A_2A_R and CD26 are not represented as homodimeric proteins.

We previously reported that ADA mutations nearly to the catalytic site that reduce the enzymatic activity, as Leu58Ala and Leu62Ala ADA mutants, also reduce the capacity of ADA to interact with A_2A_R. For these mutants, “changes detected on both *k*_cat_ and *K*_M_ values indicate that both the substrate affinity and the maximum velocity were decreased, suggesting that these mutations alter the structure of the catalytic pocket” (Gracia et al., 2013a). This was corroborated by much greater value obtained for these mutants in the affinity of the competitive structural analog purine riboside, compared to the wild type (**Table 2**). The specific enzyme activity of Leu58Ala and Leu62Ala ADA mutants is highly reduced respect to the wild-type enzyme and both mutants are unable to significantly affect agonist binding to A_2A_R (**Table 2** and Gracia et al., 2013a). Here we tested if these ADA mutants are able to induce NanoBRET signal between A_2A_R-Nluc-spacer expressing cells and CD26-YFP expressing cells. HEK-293T cells transfected with A_2A_R-Nluc-spacer (expressing 120.000 bioluminescence units) were mixed with HEK-293T cells transfected with CD26-YFP (expressing 25.000 fluorescence units) and were incubated with medium (0), with human wild-type ADA or with Leu58Ala or Leu62Ala ADA mutants previously to detect the NanoBRET signal. Positive NanoBRET signal was not detected with Leu58Ala or Leu62Ala ADA mutants, whilst NanoBRET was significantly detected with wild-type ADA (**Figure 4D**). All these results show that ADA could act as a bridge simultaneously interacting with A_2A_R and CD26 expressed in different cells, and allowing cell-cell contacts as schematized in **Figure 5**.

**Table 2:** Comparison of steady-state kinetic and agonist binding parameters of wild-type and representative ADA mutants.

Enzyme	Specific activity (μmol min^-1^ mg^-1^)^#^	*k*_cat_/*K*_M_ (M^-1^ s^-1^)^&^	*K*_i_ (PR) (μM)^&^	Δ [^3^H]CGS 21680 binding (%)^&^	EC50 (ng/ml)^&$^
WT	3 ± 1	7.3 × 10^6^	13 ± 2	88 ± 10	7 ± 3
L58A	ND	0.051 × 10^6^	>1000^∗∗^	0^∗∗^	>1500^∗∗^
L62A	ND	0.074 × 10^6^	>1000^∗∗^	0^∗∗^	>1500^∗∗^

*Wild-type and mutant enzymes were partially purified as indicated in Materials and Methods. WT, control wild-type ADA; PR, purine riboside; ND, not detected. ^#^Specific activity was determined using the substrate concentration that gives *V*_*max*_, and protein concentration was measured by the bicinchoninic acid method. ^&^Data adapted from Gracia et al. (2013a). ^*$*^EC_*50*_ value is the amount of wild-type or mutant ADA that is able to produce the 50% of the maximum increase in [^*3*^H]CGS 21680 binding to A_*2A*_R. Values are mean ± SEM of three separate experiments. ^∗∗^*P* < 0.01 against WT; statistical differences were evaluated using one-way ANOVA followed by a Dunnett’s multiple comparison *post hoc* test.*

## Discussion

Intracellular adenosine is an important intermediary metabolite, which acts as a piece in the assembly of nucleic acids and as a component of the molecule that provides the biological energy ATP (Chen et al., 2013). On the other hand, extracellular adenosine plays an important role in intercellular signaling by binding to ARs on the cell surface. This affects various physiological functions, such as cardiovascular, neurological, and immunological systems (Ohta, 2016). Most of the extracellular adenosine comes from the release and metabolism of adenine nucleotides such as ATP after several stimuli, which include inflammation, mechanical stress, tissue injury and osmotic challenge (Sun and Huang, 2016). This extracellular adenosine is degraded by ecto-ADA, which requires cell-surface anchoring proteins to stay joined to the plasma membrane. To date, four ADA-binding proteins have been described: the multifunctional CD26 protein, and the subtypes A_1_R, A_2A_R and A_2B_R of ARs.

**FIGURE 6:**
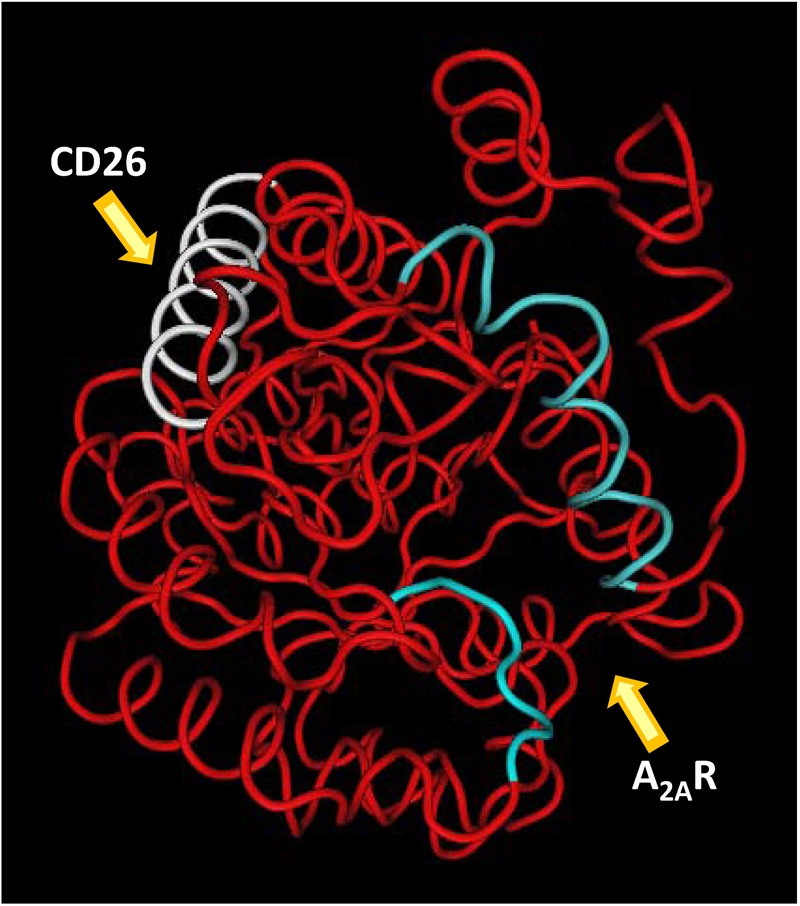
Representation of ADA regions involved in the interaction with A_2A_R and CD26. ADA in red (MMDBID:75950) was drawn with Cn3D4.1 program (http://www.ncbi.nlm.nih.gov). Helix α-1 (portion P55-I72) and loop (G184-P189) of A_2A_R binding site are in blue; helix α-2 (CD26 binding site, P126-D143) is in white.

Weihofen et al. (2004) crystallized the complex constituted by bovine ADA and human CD26 ectodomain and showed that each CD26 dimer binds two ADA molecules. In this structure two different interactions contribute to stabilize the CD26/ADA complex. In one, the Ile287-Asp297 loop A of CD26 and the Arg76-Ala91 helix α1 of ADA interact; in the other, the Asp331-Gln344 loop B of CD26 interacts with the Pro126-Asp143 helix α2 of ADA (see **Figure 6** and Cortés et al., 2015). Moreover, the crystal structure shows the intermolecular links in a highly amphiphilic interface that contributes to the CD26/ADA complex formation and also stabilizes the binding interface, where two hydrophobic loops protruding from the β-propeller domain of CD26 interact with two hydrophilic and strongly charged α-helices of ADA. This results in a very high percentage of charged residues that are involved in this protein–protein interaction (Weihofen et al., 2004). On the other hand, in this complex, ADA does not block the active site of CD26 and conversely, binding of CD26 does not block the active site of ADA; this indicates that CD26 and ADA remain catalytically active upon the complex formation (Weihofen et al., 2004; Fan et al., 2012). It has been reported that the CD26/ADA complex is selectively expressed on Hodgkin’s and ALK-positive anaplastic large cell lymphomas (Kameoka et al., 2006). Likewise, Mandapathil et al. (2012) showed ADA activity and CD26 expression in T_reg_ cells and CD4^+^ T_eff_ cells in patients with neck and head squamous cell carcinoma. All these results put these proteins in the focus of immunological regulation and point out that the costimulatory activity of ADA could be relevant in a variety of immunological diseases (Martinez-Navio et al., 2011; Casanova et al., 2012; Anz et al., 2014; Cortés et al., 2015; Klemann et al., 2016; Naval-Macabuhay et al., 2016; Wagner et al., 2016; Aliyari Serej et al., 2017).

It has been also reported that the binding of ADA to CD26 can be relevant in the regulation of lymphocyte and epithelia cell adhesion (Ginés et al., 2002). The ability of cells to adhere to one another is a fundamental property in the evolution of multicellularity. Adhesion between two different cell types is “a complex phenomenon that requires a variety of extracellular matrix (ECM) components and proteins on the surface of the interacting cells” (Ginés et al., 2002). In this mechanism, apart from cell adhesion molecules, many other soluble cell mediators such as cytokines and components of the tissue matrix such as collagen, fibronectin, etc. play a crucial role (Akiyama, 1996; Golias et al., 2011; Horwitz, 2012; Loeser, 2014). Cell adhesion links one cell to another and to the ECM, and also allows extracellular information to be integrated with the main intracellular signaling pathways. Cell adhesion is also essential in cell communication and regulation and “becomes of fundamental importance in the development and maintenance of tissues” (Khalili and Ahmad, 2015). Ginés et al. (2002) hypothesized that the ADA-CD26 module would be important for the interaction of lymphocytes with epithelial and other cell types expressing ecto-ADA in the first steps of cell to cell recognition, and it would subsequently contribute, by signaling, to the engagement of the mechanism required to change integrins over to their active conformations in T cells (Baker et al., 2009; Zhang and Wang, 2012; Moretti et al., 2013; Meli et al., 2016).

Adenosine receptors A_1_R, A_2A_R, and A_2B_R are the second type of ecto-ADA binding proteins (see section “Introduction”). Among these receptors, A_2A_Rs are highly expressed in spleen, thymus, blood platelets, striatum, olfactory tubercle and expressed to a lesser extent in the heart, lung, blood vessels and other brain regions including cortex and hippocampus (Cunha et al., 1997; Chen et al., 2013; Cortés et al., 2015; Stockwell et al., 2017). A_2A_Rs are expressed on most immune cells, including T cells, NK and invariant natural killer T cells, DCs, macrophages, monocytes, mast cells, eosinophils and B cells (Cekic and Linden, 2016). The A_2A_R is recognized as mediating major adenosine anti-inflammatory activity (Welihinda and Amento, 2014) and is involved in various metabolic and pathological states including sleep regulation, ischemia-reperfusion injury, inflammation and autoimmune diseases and neurodegenerative disorders (Borea et al., 2016; Sharma et al., 2016; Lazarus et al., 2017; Stockwell et al., 2017). Likewise, A_2A_R is responsible for most of the known immunoregulatory effects of adenosine in the immune system and is a molecule crucially involved in CNS autoimmunity (Ingwersen et al., 2016).

Bioluminescence resonance energy transfer has provided much of the evidence supporting GPCRs oligomerization. Angers et al. (2000) used this technique in HEK-293 cells to demonstrate that human beta-2 adrenergic receptors form constitutive homodimers. We and others demonstrated that A_1_R and A_2A_R are expressed as homodimers or higher-order oligomers, which are the functional species *ex vivo* or in transfected cells (Ciruela et al., 1995; Canals et al., 2004; Briddon et al., 2008; Gandia et al., 2008; Vidi et al., 2008; Namba et al., 2010; Gracia et al., 2011, 2013b; Casadó-Anguera et al., 2016; Navarro et al., 2016). For both receptors, enzymatically active or Hg^2+^-inactivated ADA increases the signaling and the receptor affinity by a protein–protein interaction. ADA acts as an allosteric modulator of A_1_R and A_2A_R, altering their quaternary structure and, consequently, their pharmacological and functional characteristics (Ciruela et al., 1996; Saura et al., 1996; Sarrió et al., 2000; Sun et al., 2005; Gracia et al., 2008, 2011, 2013b). The ADA-induced molecular rearrangement in the corresponding receptor structure, demonstrated by BRET experiments, is in good agreement with the ADA-promoted increase in agonist-induced signaling and ligand affinity for both A_1_R and A_2A_R. These results suggest that ADA can exert “a finely tuned modulation of adenosine neuroregulation that may have important implications for the function of neuronal ARs” (Cortés et al., 2015).

In the last decade, the only high-resolution information available for the ARs comes from structures of A_2A_Rs complexes with several agonists, antagonists, an inverse agonist and an engineered G protein (Jaakola et al., 2008; Doré et al., 2011; Lebon et al., 2011, 2015; Xu et al., 2011; Congreve et al., 2012; Liu et al., 2012; Hino et al., 2012; Carpenter et al., 2016; Segala et al., 2016; Cheng et al., 2017; Sun et al., 2017). These structures can facilitate the discovery of more effective and selective A_2A_R ligands and have provide a detailed molecular understanding of the receptor function and the conformational landscape between the agonist and the antagonist states of this receptor (Bertheleme et al., 2014; Cheng et al., 2017). By a combination of computational docking simulations and molecular dynamics simulations between the crystal structures of human ADA and human A_2A_R, we demonstrated that the putative molecular regions of ADA involved in the interaction with the A_2A_R were opposed to the ADA domains interacting with CD26 (see above) (Gracia et al., 2013a). We now demonstrate a direct molecular interaction between ARs, specifically A_2A_R, and CD26 bound by ADA, using biophysical techniques. To achieve this goal it is necessary an energy transfer at the extracellular level between two transmembrane proteins that only happens when they are linked by ADA. We have taken advantage of a variant of the BRET assay in which, using fusion proteins of the Nluc enzyme, both the translocation of the N-terminal fusion protein and the steric hindrance are improved (Machleidt et al., 2015; Mo and Fu, 2016). This is compatible with a macromolecular complex in which A_2A_R and CD26 are bridged by ADA (see **Figure 5**), in a narrow range of ADA concentrations around the binding affinity values (Saura et al., 1996; Gonzalez-Gronow et al., 2004), and showing a peak pattern, instead of a saturable pattern. Lower concentrations are insufficient to bridge the trimer and higher ADA concentrations favor the dimeric ADA-CD26 and ADA- A_2A_R complexes (see **Figure 4A**, top panels). Soluble CD26 could interfere with this role of ADA in many pathological conditions, such as obesity and several viral infections, where its concentration is highly increased, but not in healthy physiological conditions, where its concentration (up to 4 nM) is much lower than its affinity for ADA (see Yu et al., 2011). Because A_2A_R and CD26 are homodimers (see above), their protein arrangement is probably more complex, with two ADA molecules linking the two homodimers.

Gracia et al. (2013a) highlighted the contribution of the 55–65, 114–118, 155–158, and 184–189 amino acidic segments of ADA to the A_2A_R/ADA interface. The 55–65 stretch interacts with the extracellular loop 2 of the receptor whereas the 184–189 stretch interacts with the N-terminus of the A_2A_R (see **Figure 6**). Moreover, these two stretches constitute the structural gate to the catalytic site of ADA in the tertiary structure of this enzyme, which can take different conformations: the closed and the open forms (Wilson et al., 1991; Kinoshita et al., 2005). In the absence of the substrate adenosine, ADA adopts the open form, whereas in complexes with adenine-based substrate analogs it adopts the closed form, which indicates that it is obtained after the binding of the substrate (Cortés et al., 2015; Maiuolo et al., 2016). Since ADA can increase the binding of the ligand to ARs in the absence of adenosine, it was suggested that the open form, and not the closed one, is able to bind to A_2A_R (Gracia et al., 2013a; Cortés et al., 2015). In fact, using alanine scanning mutagenesis we showed that the two amino acid regions that participate in the structural gate of the active site pocket (the α-1 helix 55–65 and the 184–189 loop) play a central role in ADA catalysis and/or ADA-induced modulation of agonist binding to A_2A_R (Gracia et al., 2013a). In particular, we showed that mutations of the hydrophobic residues Leu58 and Leu62 produce a 100-fold decrease of the catalytic efficiency (see **Table 2**), because they decrease both the maximum velocity and the substrate affinity. This suggests that hydrophobicity may help to maintain the control of the catalysis and the affinity for adenosine (Gracia et al., 2013a). Moreover, when we performed experiments with increasing concentrations of these ADA mutants to determine the amount of enzyme able to produce an increase of the 50% of the maximum agonist (CGS) binding (EC_50_ values), we obtained a very big increase (>200-fold) in the EC_50_ values of Leu58 and Leu62 with respect to the ADA wild type (see **Table 2**). These results point out that the α-1 helix is an important ADA domain involved in the allosteric modulation of the A_2A_R. For this reason, in our results, NanoBRET is abolished when these mutants are used to link cell populations with A_2A_R and with CD26.

Besides the existence of the binary complexes between ADA-ARs and ADA-CD26, higher order protein aggregates containing both ARs and ADA have been postulated. In that sense, Franco et al. (1997, 1998) suggested that ecto-ADA may participate in cell to cell contacts (CD26/ADA/CD26; CD26/ADA/A_1_R and A_1_R/ADA/A_1_R) which can be of relevance in neural functionality and development. Later, Torvinen et al. (2002) proposed the existence of functional trimeric complexes formed by ADA and A_1_R and dopamine D_1_ receptors in cortical neurons and that their aggregation can be modulated by both adenosine and dopamine. Likewise, by acting as a bridge between A_2B_R on DCs and CD26 on T cells, by forming an “immunological synapse” (Pacheco et al., 2005; Franco et al., 2007), ADA acts as a costimulatory molecule in T-cell-DC co-cultures enhancing Th-1/pro-inflammatory cytokine secretion, T-cell proliferation, and T-CD4^+^ cell activation, memory, and Foxp3^+^ generation in healthy subjects, but also in subjects infected with HIV (Martinez-Navio et al., 2011; Casanova et al., 2012). Our results with NanoBRET assays reinforce these hypotheses and extend them to the formation of CD26/ADA/A_2A_R complexes, where ecto-ADA anchored to CD26 could direct the interaction between T cells and ARs-containing cells, such as neurons, DCs, and so on. Future studies demonstrating adhesion between cells promoted by ADA (e.g., DCs and T-cells) under physiological conditions, and analyzing how this ternary complex affects the function of the three proteins involved, will be necessary.

## Conclusion

In this study, using biophysical techniques we demonstrate the possibility of formation of the ternary complex CD26/ADA/A_2A_R. This molecular interaction is specific, as it is abolished by a human-specific mAb against CD26, TA5.9-CC1-4C8, or by ADA mutants (Leu58Ala and Leu62Ala) with highly reduced capacity to interact with A_2A_R. The bridge in the ternary complex is neither produced by a non-specific protein as albumin or between A_2A_R and another membrane protein different of CD26, as NMDA receptor, newly showing the specificity of the ADA-linked proteins. In that ternary complex, ADA can act as a bridge that interacts simultaneously with A_2A_R and CD26 expressed in different cells. This fact could allow a physiological cell-cell adhesion between, for example, DCs or neurons that express A_2A_R (Fredholm et al., 2005; Cekic and Linden, 2016) and T cells that express CD26 (Klemann et al., 2016). This would add a new metabolic function for ecto-ADA, that being a single chain protein it has been considered as an example of moonlighting protein (Cortés et al., 2015). This is because it performs more than one functional role (Copley, 2012, 2014; Jeffery, 2014, 2015, 2016; Chapple and Brun, 2015): (1) as a catalyst that degrades adenosine; (2) as a costimulatory molecule promoting T-cell differentiation and proliferation by interacting with CD26; (3) as an allosteric modulator of A_1_R and A_2A_R, without portioning these functions in different subunits; and (4), as a bridge, forming cell-to-cell contacts, as described in the present study.

## Author Contributions

EM, JC, EG, AC, and VC performed the experiments and analyzed the data. EM, JM, CL, AC, and VC designed the experiments. EM, EG, EC, AC, and VC wrote the manuscript.

## Conflict of Interest Statement

The authors declare that the research was conducted in the absence of any commercial or financial relationships that could be construed as a potential conflict of interest.
